# Monofunctional Platinum(II) Anticancer Agents

**DOI:** 10.3390/ph14020133

**Published:** 2021-02-07

**Authors:** Suxing Jin, Yan Guo, Zijian Guo, Xiaoyong Wang

**Affiliations:** 1 State Key Laboratory of Coordination Chemistry, School of Chemistry and Chemical Engineering, Nanjing University, Nanjing 210023, China; jinsuxing@nju.edu.cn (S.J.); 20191022@hncj.edu.cn (Y.G.); zguo@nju.edu.cn (Z.G.); 2 School of Materials and Chemical Engineering, Henan University of Urban Construction, Pingdingshan 467036, China; 3 State Key Laboratory of Pharmaceutical Biotechnology, School of Life Sciences, Nanjing University, Nanjing 210023, China

**Keywords:** anticancer drug, drug design, metal-based drug, monofunctional platinum complex

## Abstract

Platinum-based anticancer drugs represented by cisplatin play important roles in the treatment of various solid tumors. However, their applications are largely compromised by drug resistance and side effects. Much effort has been made to circumvent the drug resistance and general toxicity of these drugs. Among multifarious designs, monofunctional platinum(II) complexes with a general formula of [Pt(3A)Cl]^+^ (A: Ammonia or amine) stand out as a class of “non-traditional” anticancer agents hopeful to overcome the defects of current platinum drugs. This review aims to summarize the development of monofunctional platinum(II) complexes in recent years. They are classified into four categories: fluorescent complexes, photoactive complexes, targeted complexes, and miscellaneous complexes. The intention behind the designs is either to visualize the cellular distribution, or to reduce the side effects, or to improve the tumor selectivity, or inhibit the cancer cells through non-DNA targets. The information provided by this review may inspire researchers to conceive more innovative complexes with potent efficacy to shake off the drawbacks of platinum anticancer drugs.

## 1. Introduction

**Figure 1:**
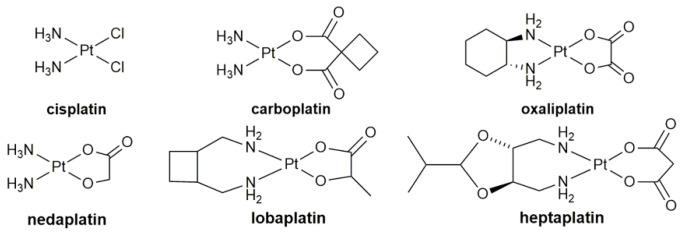
Chemical structures of clinically approved platinum anticancer drugs.

Cisplatin and its analogues, carboplatin, oxaliplatin, nedaplatin [1], lobaplatin [2], and heptaplatin [3] (Figure 1) have been approved for clinical use in different countries to treat multiple solid neoplasms, and approximately half of the chemotherapy strategies include platinum drugs [4,5,6]. However, these drugs are structural congeners of cisplatin and therefore, some drawbacks are inherited [7,8,9]. For instance, DNA is believed to be the ultimate target of cisplatin, so are other platinum drugs [10,11,12]. Nevertheless, DNA damages could be easily repaired by DNA repair mechanisms. Therefore, all the existing platinum anticancer drugs encounter drug resistance [13,14]. Moreover, their nonspecific accumulation in the hypermetabolic state tissues results in the systemic toxicity.

One strategy for increasing the potency while mitigating the side effects of platinum complexes is to exploit new compounds that operate on novel mechanisms [15,16]. In this respect, cationic monofunctional Pt^II^ complexes that contain only one labile ligand exhibit special anticancer activities in comparison with cisplatin analogues due to the changes in DNA-binding mode, cellular accumulation, and even the mechanism of action [17,18]. These complexes represent an alternative class of anticancer agents that violate the classical structure-activity relationships (SAR) of platinum complexes [15]. Their antineoplastic activity arises from diverse interactions with different biomolecules and displays a distinct spectrum of activity in favor of circumventing the drug resistance or side effects [19,20,21,22].

DNA remains the major, if not the only, target for most of monofunctional Pt^II^ complexes. Nevertheless, the DNA-binding mode or process is different from that of cisplatin and its analogues. Each monofunctional complex could form at most one covalent bond with the N7-guanine on the DNA strands rather than two covalent Pt−DNA cross-links as cisplatin does [23]. The earliest prototype complexes [Pt(NH_3_)_3_Cl]^+^ and [Pt(dien)Cl]^+^ (dien = diethylenetriamine) are thought to be inactive towards cancer cells, since according to the prevailing view only neutral and square-planar Pt^II^ complexes with a pair of inert ligands in a *cis*-configuration possess anticancer activity [6,24,25]. However, the preconceived belief was overturned by the finding that *cis*-[Pt(NH_3_)_2_(Am)Cl]^+^ (Am is an aromatic *N*-heterocyclic amine) inhibited tumor cells in vitro and leukemia (L1210 and P388) in mouse models [26], where Pt^II^ formed stable Pt–DNA adducts and the complex intercalated into DNA. Further studies found that amino or Am groups could lose upon binding to DNA, thereby achieving a bifunctional coordination [27].

**Figure 2:**
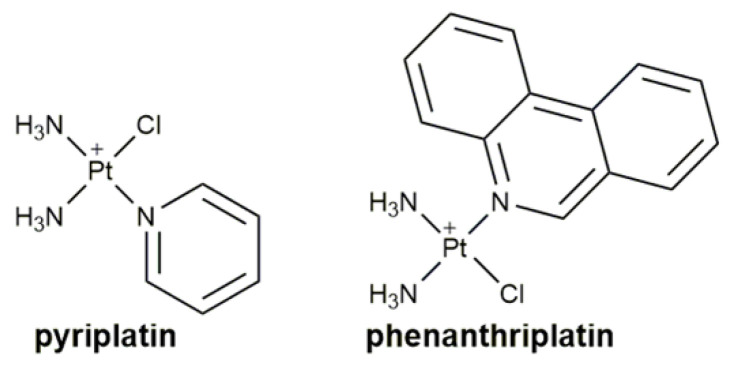
Chemical structures of pyriplatin and phenanthriplatin.

Afterwards, it was found that the cationic Pt^II^ complex pyriplatin (Figure 2) only formed a monofunctional adduct with DNA and induced little distortion in the DNA double helix upon binding. In addition, organic cation transporters (OCTs) were involved in its cellular uptake and activity [28,29]. SAR studies indicated that the steric hindrance of the pyridine ligand played an important role in regulating the action of RNA polymerase II [30,31,32]. Phenanthriplatin (Figure 2) also formed monofunctional adducts with guanine bases as well as duplex DNA once the chloride ligand lost. Phenanthriplatin-DNA adducts generate steric hindrance in the major groove of DNA and thus, stall the progression of RNA polymerase II on the damaged DNA templates and inhibit DNA polymerases [33]. This complex has a unique cytotoxic profile as it was 7–40 times more active than cisplatin in many human cancer cell lines and, unlike pyriplatin, showed an activity spectrum in the more extensive NCI-60 panel of cell lines [34,35]. Other monofunctional Pt^II^ complexes that suppressed RNA polymerase II and DNA synthesis, and displayed high cytotoxicity against cancer cells were also reported [36,37,38,39]. Yet, this is not the whole story on the mechanism of action. More and more studies revealed that the target of monofunctional Pt^II^ complexes is not limited to or even relevant to DNA. Therefore, the established SARs no longer fit them. This review will introduce some representative monofunctional Pt^II^ complexes published in the past 5 years or so and discuss their mechanism of action if possible.

## 2. Fluorescent Monofunctional Pt^II^ Complexes

DNA has been extensively studied as the ultimate cellular target of platinum complexes, while much of the mechanism of action still remains unknown. Although some small ions or molecules, amino acids, peptides, and proteins are thought to be implicated in the mechanism, the details on the cellular interactions are largely unclear. Therefore, it is of great significance to study the behavior of Pt^II^ complexes in cancer cells at the molecular level. Tethering fluorophores to the Pt^II^ center of the complexes could form fluorescent molecules, by this means the cellular location of the complexes could be mapped through fluorescence imaging.

**Figure 3:**
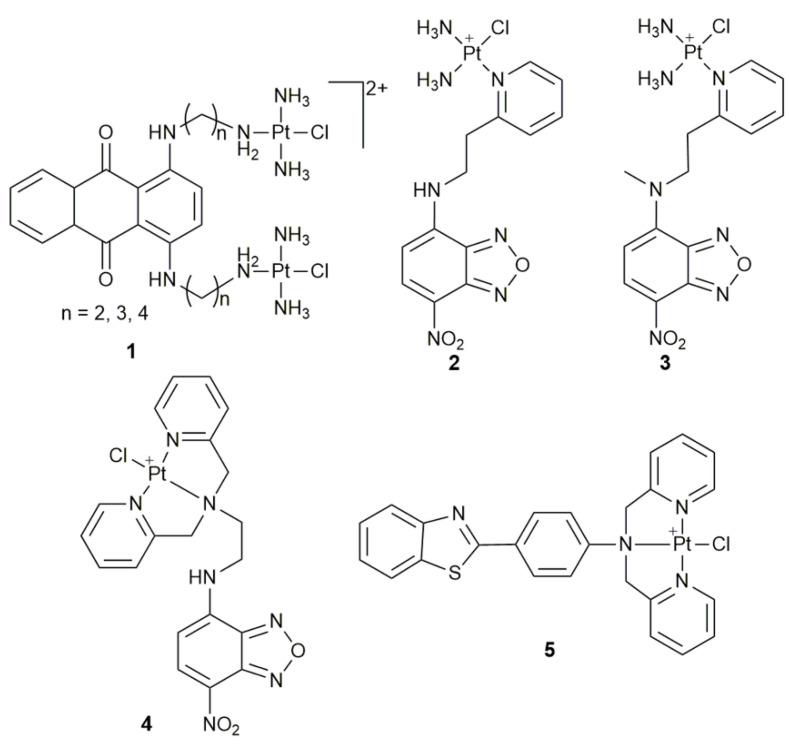
Chemical structures of complexes **1**–**5**.

Dinuclear Pt^II^ complex **1** incorporate a fluorescent anthraquinone intercalator in the structure (Figure 3). Its major merit is to monitor the subcellular localization by fluorescence microscopy on account of the innate fluorescence of the intercalator. Complex **1** exhibited high cytotoxicity in the U2-OS cell line (the designation of U2-OS and those of other cell lines appeared hereafter are listed in Appendix A Table A1 at the end of the article) and overcame resistance in the cisplatin-resistant U2-OS/Pt cell line. Their cellular process in both cell lines was similar, which may be due to the formation of intercalative DNA-adducts that could evade the DNA repair mechanism responsible for removing the cisplatin adducts [40]. The fluorescence indicated that **1** rapidly entered the U2-OS cells and accumulated in the nucleus, thereby reaching the biological target of the Pt and intercalating moieties—DNA [41,42]. The Pt moiety was excreted from the cell via the Golgi apparatus, while the weakly basic anthraquinone ligand accumulated in the Golgi complex, where it was taken up by lysosomes and then transported to the cell surface. Interestingly, contrasting results were found in A2780 cells, implying that different cell lines may respond to Pt drugs differently [41]. In cisplatin-resistant A2780 cells, the complexes were sequestered into lysosomes and displayed cross-resistance with cisplatin.

Fluorescent complexes **2** and **3** (Figure 3) were used to track their cellular distribution via detecting the fluorescence, thus providing new insights into the mechanism of action [43]. Complex **3** is more suitable for cellular imaging than **2**. Particularly, in contrast to the rapid entry to cells but inaccessibility to the nucleus for the ligand, cationic **3** entered the HeLa cells slowly and mainly accumulated in the nucleoli. It bound to the cytoplasmic vacuoles, resulting in a different distribution pattern from that of neutral fluorescent Pt complexes. Complex **3** not only acted as a probe to trace its cellular behavior, but also induced non-apoptotic cell death. Similarly, complex **4** (Figure 3) realized the in vitro and vivo fluorescence imaging [44]. Its cellular uptake was much slower than that of the ligand and it could get into the nucleus, suggesting that the Pt^II^ center played an important role in reducing the uptake process and promoting its affinity for DNA. Complex **4** also exhibited preferential affinity for mitochondria. 

Another cytotoxic fluorescent complex **5** (Figure 3) constructed by tethering a fluorophore thioflavin-T (ThT) derivative to the 3N-chelated Pt^II^ center was suitable for cellular imaging in living cells [45]. Fluorescence imaging showed that **5** was sequestrated in mitochondria and acidic lysosomes after slow entry to the cells. The finding provides new insights into the cellular distribution of positively-charged monofunctional Pt^II^ complexes. It should be noted that in analyzing the distribution of fluorescent complexes, a comparison with the distribution of free ligands is necessary so as to eliminate the false fluorescence emitted by the dissociated fluorophores.

## 3. Photoactive Monofunctional Pt^II^ Complexes

The toxic side effects resulted from the non-specific accumulation of Pt anticancer agents hinder their broader application in clinical treatment. Photoactive Pt complexes offer an opportunity to develop new anticancer drugs responsive to light [46,47]. Photodynamic therapy (PDT) is a minimally invasive method that produces selective cytotoxicity to malignant tumor cells. It has been used to treat different tumors, such as bladder and prostate cancers [48]. The anticancer effect of PDT is achieved by killing cancer cells through reactive oxygen species (ROS) such as singlet oxygen (^1^O_2_) produced by photosensitizers (PSs) under light irradiation [49]. Non-irradiated PSs generally have low dark toxicity, high ^1^O_2_ quantum yield, and good cellular accessibility [50], while the irradiated one is strongly bioactive. It has been shown that the combination of Pt complexes with PSs could produce synergistic effects [6,51,52].

**Figure 4:**
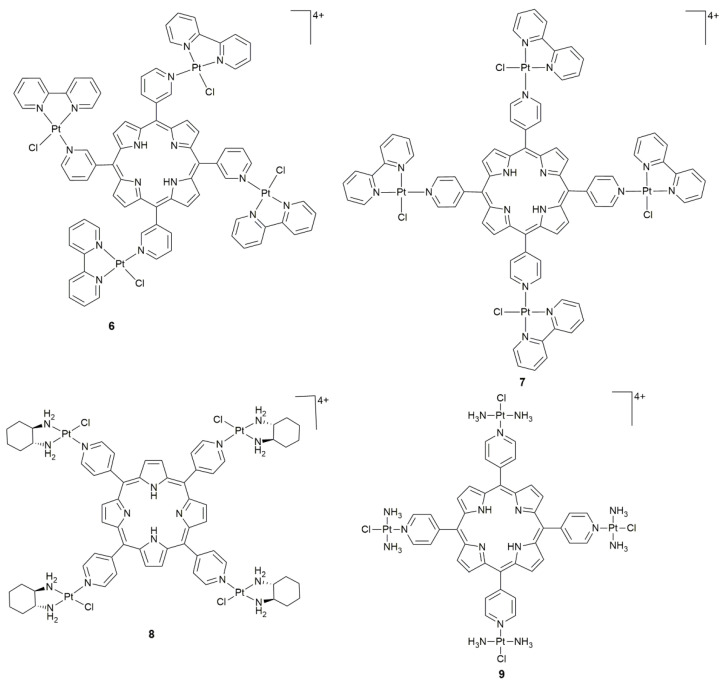
Chemical structures of complexes **6**–**9**.

Porphyrins are representative PSs for PDT owing to the ring structure with 18 conjugated π electrons, which endow the compounds with photophysical properties and selective retention or accumulation in tumors due to preferential binding to low density lipoproteins [53,54]. The structure of porphyrin is susceptible to functionalization of surrounding substituents, especially the presence of metal complexes around or in the core could modify the local environment, improving the solubility or introducing specific functionalities [54,55]. In order to potentiate the action of these structures, conjugations with Pt^II^ complexes were tried. The isomeric *tetra*-cationic(pyridyl)porphyrin Pt^II^ complexes **6** and **7** (Figure 4) possessed cytotoxicity against metastatic WM1366 cells under white-light irradiation, inducing apoptosis via the activation of caspase-3 and -9 and alteration of cell cytoskeleton actin [56]. In silico study indicated that these complexes could be employed to deliver drugs owing to the affinity to the N-terminal region of ApoB-100.

Complex **8** (Figure 4) showed an excellent photocytotoxicity (50 W LED light, 6 J cm^−2^, 30 min) due to the high ^1^O_2_ quantum yield, nuclear internalization, and a caspase-3-induced apoptosis with negligible dark toxicity [57]. Oxaliplatin was chosen as the pharmacophore since its DNA binding rate is faster than that of cisplatin. Moreover, it could improve the hydrophilicity and eliminate the formation of aggregates by increasing the electrostatic repulsion from charged functional groups [58]. Furthermore, **8** completely wiped out the tumor tissue in colon26 tumor-bearing mice. Similarly, the aqueous solubility, cellular uptake, and photophysical property of the tetraplatinated porphyrin complex **9** (Figure 4) were improved by incorporating Pt^II^ moieties [51], which directed the porphyrin to the nucleus and enhanced the nuclear Pt accumulation. The binding to DNA involves both covalent bonding with N7-guanine by Pt^II^ and intercalation by the porphyrin unit. Complex **9** demonstrated a promising photocytotoxicity with extremely high toxicity towards human cancer cell lines upon irradiation (6.95 J cm^−2^, 420 nm, 15 min, HeLa: IC_50_ = 37 nM; A2780: IC_50_ = 21 nM; CP70: IC_50_ = 19 nM), and a phototoxic index up to 5000 in the cisplatin-resistant CP70 cell line.

**Figure 5:**
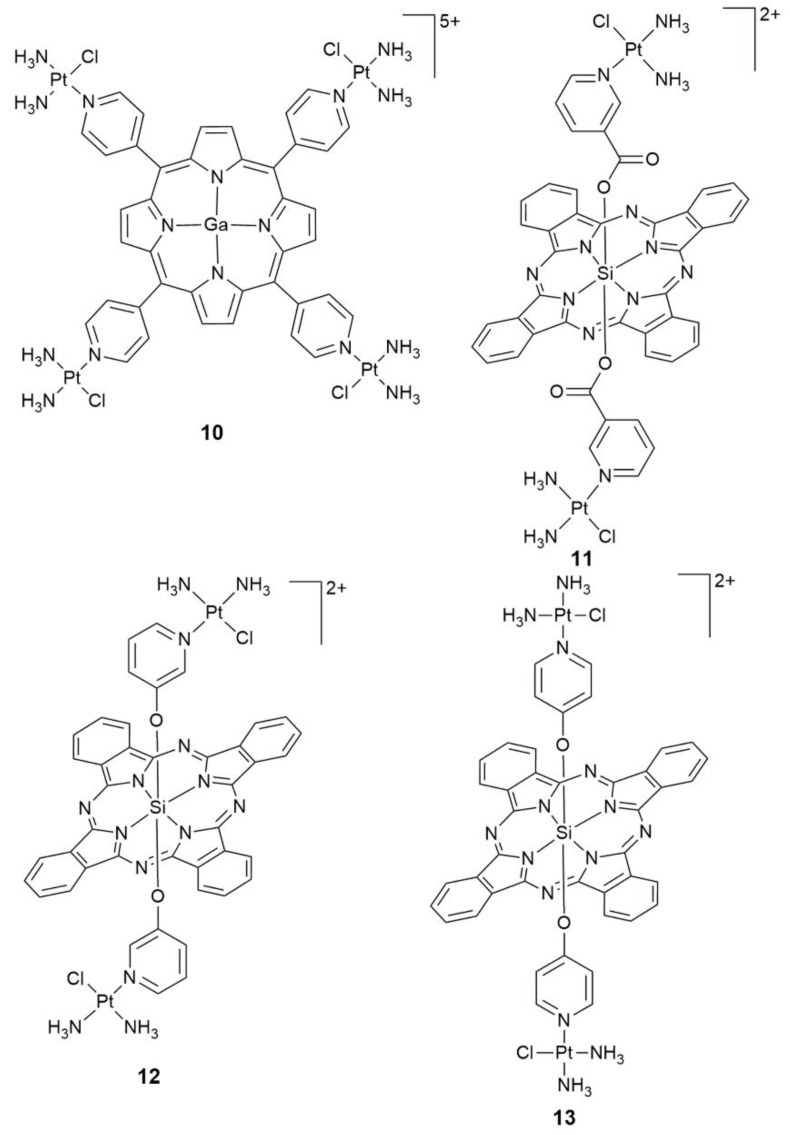
Chemical structures of complexes **10**–**13**.

Despite the fact that porphyrin-based Pt complexes have many advantages, the poor solubility and aggregation of porphyrins affect their cellular uptake and limit their activities and applications in vivo. Thus, water-soluble tumor-targeted PS **10** (Figure 5) with a porphyrin framework containing Ga^III^ and Pt^II^ moieties was developed [59]. Complex **10** is an efficient ^1^O_2_ generator owing to the heavy atom effect, acidic pKa, and localization in cytosol. It showed negative dark cytotoxicity due to the larger hydrophilicity, slower and lower cellular uptake. Moreover, it exhibited remarkable photocytotoxicity and interaction with DNA, accumulated in tumor prominently (tumor/muscle ratio > 9), and inhibited tumor growth almost completely over 2 weeks. No significant systemic toxicity including weight loss and adverse reactions were observed.

The low efficiency of ^1^O_2_ generation within the maximum tissue penetrating and biocompatible spectral window (650–850 nm) is another limitation in addition to the aggregation and solubility of PSs. Si^IV^ phthalocyanines (SiPc), characterized by reduced aqueous aggregation and high ^1^O_2_ quantum yield when illuminated with tissue-penetrating far-red light, could solve this problem [60,61]. A positively-charged Pt^II^–Si^IV^ phthalocyanine complex **11** (Figure 5) was selectively delivered to cancer cells by the hyaluronic acid (HA) formulated nanoparticles with the mediation of the CD44 receptor [62]. The nanoparticles showed improved aqueous solubility, specific uptake, photo-enhanced cytotoxicity (~1500-fold) and mitochondrial accumulation in CD44-overexpressed breast cancer cells over normal ones in red light (45 min, 660–680 nm, 5.5 ± 2.5 mW cm^−2^). Interestingly, the nanoconjugate delivered **11** only to cancer cells, which resulted in the generation of cytotoxic ^1^O_2_ and Pt^II^ species. 

Complexes **12** and **13** (Figure 5) presented a 25- and 7-fold enhancement, respectively, in cytotoxicity against HeLa cells at 1 μM under illumination with red light in comparison to those kept in the dark [63]. Both complexes demonstrated a potential to serve as DNA-targeting PDT agents. The Pt^II^ moieties lead the PDT moiety to approach DNA and execute red-light-induced oxidative damage, while the photoactive SiPc moiety endows the Pt^II^ units with a red-light-induced photochemical property that may lead to enhanced DNA platination. This design was expected to be superior over solo therapeutic modalities and obtain drugs with improved activity and reduced side effects. The use of phthalocyanine could alleviate some limitations of PDT, however, the self-aggregation in aqueous media may affect its photosensitivity. In brief, the Pt–porphyrin or Pt–phthalocyanine conjugates maintained the intrinsic properties of an individual unit in cancer cells, and thus could act as dual threat anticancer agents.

**Figure 6:**
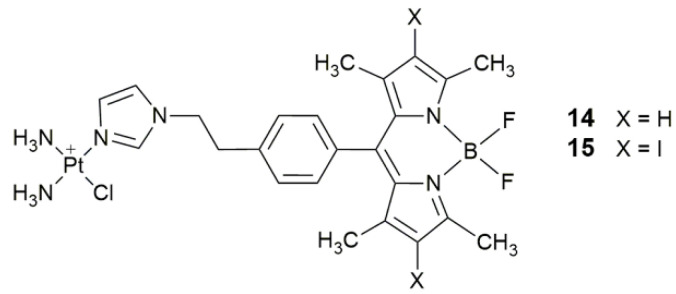
Chemical structures of complexes **14** and **15**.

PSs based on non-macrocyclic dyes were also used to design conjugates for the combinative effect of PDT and inhibition of DNA transcription. Complexes **14** and **15** (Figure 6) showed remarkable photocytotoxicity in visible light (400−700 nm, 10 J cm^−2^) to the HaCaT and MCF-7 cells, with the IC_50_ being in the nanomolar level, while they were almost nontoxic (IC_50_ > 80 μM) in the dark [64]. Complex **14** was emissive and showed significant localization in the mitochondria and minor localization in the endoplasmic reticulum (ER), and hence could be used for cellular imaging and reducing the drawbacks associated with bifunctional binding of nuclear DNA (nDNA) by Pt drugs, such as nuclear excision repair (NER).

**Figure 7:**
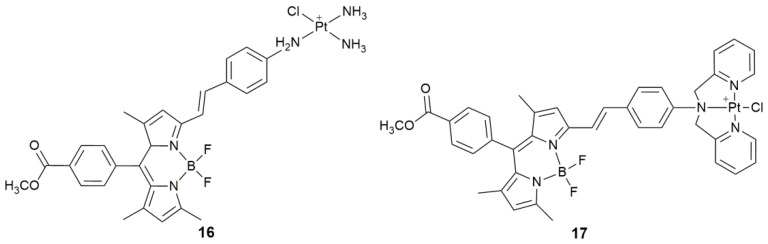
Chemical structures of complexes **16** and **17**.

Cationic PS is helpful to promote the cellular accumulation of anticancer agents and damage the cell membrane by photo-induced ROS in situ, which is considered as the main mechanism to enhance the cellular uptake [65]. Complex **16** (Figure 7) exhibited distinct anticancer cytotoxicity against MCF-7, SGC-7901, A549, and HeLa cell lines via short time photoirradiation (532 nm, 3.5 mW cm^−2^, 5 min) [66]. It first accumulated on the surface of the cell membrane in the dark for its membrane-anchoring ability, and then acted as a PS, promoting the damage to the cell membrane in situ to increase its accumulation in tumor cells. Although the molecular mechanism was not studied, short time photoirradiation seems to play a key role in activating the complex.

Lysosomes as special organelles are responsible for degrading and recycling extracellular materials via endocytosis and phagocytosis, and intracellular poisonous species via autophagy [67,68]. Growing evidence indicates that lysosomes are capable of isolating some Pt complexes to reduce their contact with nDNA, thus reducing the DNA-damaging effect [69]. Therefore, silencing Pt complexes in lysosomes and then activating them specifically in the tumor tissue might be a method for improving the antitumor activity and alleviating side effects. Complex **17** (Figure 7) is the first example of photoactive monofunctional Pt^II^ complex capable of lysosomal escape [70]. It was sequestered in lysosomes via endocytosis and showed low cytotoxicity to both normal and tumor cells without photoirradiation. Interestingly, it escaped from the lysosomes to the nucleus upon short-time photoirradiation (532 nm, 3.5 mW cm^−2^, 5 min) due to the photoinduced ability to generate ROS. Apart from damaging lysosomes to release **17** into the cytosol and nucleus, ROS also decreased intracellular GSH levels to impede its deactivation in the cytosol and further increased its accessibility to nDNA favorable for the antitumor activity.

**Figure 8:**
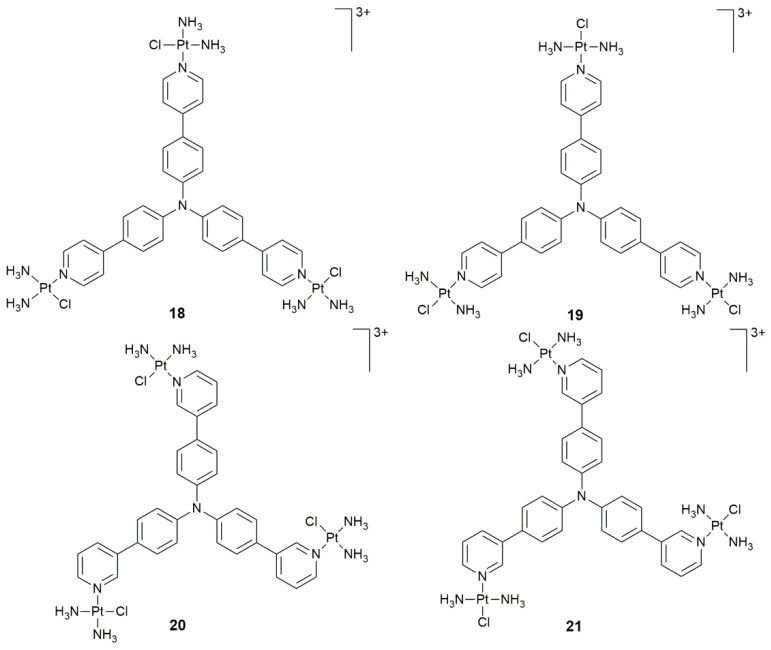
Chemical structures of complexes **18**–**21**.

Pt complexes combined with PSs may exhibit synergistic effects, but most of them are limited to Pt–porphyrin conjugates. Replacing porphyrin with other PSs is another way to develop effective PDT agents. A series of DNA-binding Pt^II^–triphenylamine complexes were developed as potential PDT anticancer agents [71,72,73,74]. The fluorogens π-conjugated triphenylamines, owing to two-photon absorption and aggregation induced emission (AIE) properties, are used as fluorescent probes or theranostic agents [75]. In order to improve the PDT efficiency of the conjugates and systematically investigate the anticancer SAR, trinuclear Pt–triphenylamine isomers **18−21** (Figure 8) were developed [76]. Complexes **18** and **19** exhibited much better PDT activity than complexes **20** and **21** owing to the redder absorption and emission wavelength, higher cellular uptake and ^1^O_2_ quantum yield, stronger DNA-binding and photo-cleavage ability. In addition, complexes **18** and **19** mainly accumulated in the nucleus, while complexes **20** and **21** distributed mainly in the cytoplasm. Particularly, complex **19** elicited DNA damage responses, arrested the cell cycle in the G2/M phase, and led to apoptosis in cancer cells upon light irradiation at 425 nm (40 mW cm^−2^, 15 min). Moreover, it exhibited significant PDT effect on HeLa xenograft-bearing mice, including reduction in the tumor volume and cell death in tumor sections, but showed no noticeable side effects on body weight and major organs.

## 4. Targeted Monofunctional Pt^II^ Complexes

Although Pt-based drugs play an important part in cancer therapeutic regimens, their widespread use is still limited by the severe toxic side effects arising from the lack of selectivity for cancer cells. To overcome this defect, cancer-targeted Pt complexes are developed. The targeting group(s) in such complexes could direct the Pt warheads to cancer cells by interacting with the receptors overexpressed on the cell surface [77], or direct to the tumor as a whole through the interaction with the tumor-related cell surface markers such as antigens or receptors [78]. Targeting could also be achieved at the subcellular level, allowing Pt to be directed to specific organelles to elicit biological effects [6,78]. Herein, we particularly focus on monofunctional Pt^II^ complexes designed for these purposes.

**Figure 9:**
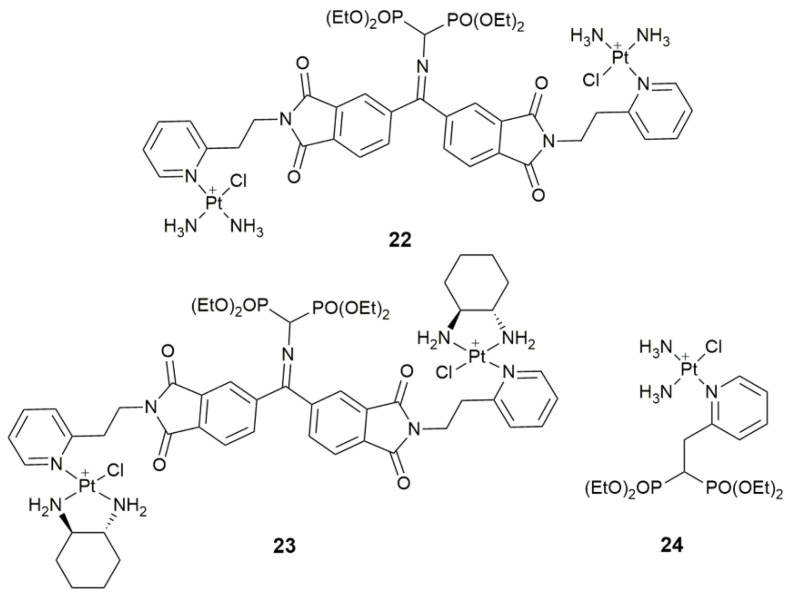
Chemical structures of complexes **22**–**24**.

Taking osteosarcoma (OS) as an example, which is a primary malignant bone tumor severely threatens the life of adolescents [79]. Owing to the peculiar tumor sites (knee joint and appendicular skeleton) and lack of knowledge about driving oncogenes, as well as insufficient drug concentration in the tumor site, OS is extremely difficult to treat [80,81]. Cisplatin is ineffective for OS due to its poor accessibility and severe systemic toxicity [82]. The coordination of phosphonate groups with Ca^2+^ ions endows bisphosphonates with a special affinity for hydroxyapatite in the bone matrix [83]. Complexes **22** and **23** (Figure 9) bearing a bone-targeting bisphosphonate moiety exhibited potential selectivity for OS [84]. The cytotoxicity of cisplatin-derived **22** was higher than that of oxaliplatin-derived **23** towards the U2-OS cells. Bisphosphonate also improved the lipophilicity and cellular uptake of the complexes. Lipophilic **24** (Figure 9) was optimized to maintain the bone-targeting property as well as to minimize the reactivity of the Pt^II^ center in order to decrease the systemic toxicity [85]. Unlike complexes **22** and **23**, the molecular mechanism of complex **24** involves both DNA binding and mevalonate pathway. Its acute toxicity is 7-fold lower than that of cisplatin. The introduction of bisphosphonate provides a new possibility to overcome the ineffectiveness and systemic toxicity of Pt drugs for the treatment of OS.

**Figure 10:**
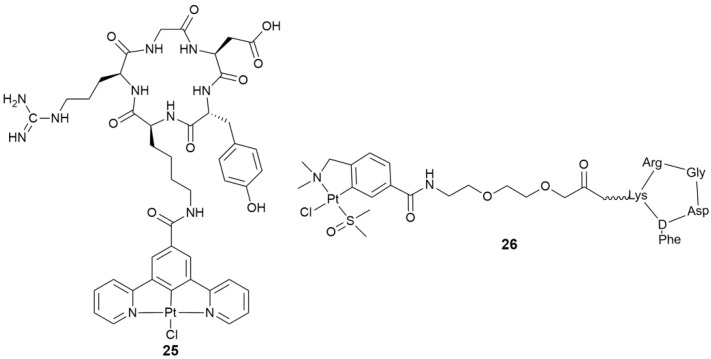
Chemical structures of complexes **25** and **26**.

Targeting the whole tumor is based on the specific expression of some receptors or antigens on the surface of tumor cells. Tumor-associated receptors are well documented in the literature, for example, transferrin, selectins, integrins, folate receptor, glucose transporter (GLUT), galectins, hyaluronic acid receptors, and the asialoglycoprotein receptor [6,86]. Targeting these receptors could selectively deliver a cytotoxic agent to cancer cells. Integrins are heterodimeric transmembrane cell adhesion glycoproteins, which play a key role in enhancing migration, invasion, and proliferation of cancer cells, and even are linked to tumor angiogenesis [87]. The synthesis and biological profile of a Pt^II^-c(RGDyK) conjugate **25** (Figure 10) for integrin-targeted PDT has been reported. Complex **25** was moderately cytotoxic towards six cancer cell lines with different levels of integrin expression [88]. It was taken up rapidly by receptor-mediated endocytosis and generated ^1^O_2_ efficiently upon irradiation, thus showing enhanced anticancer activity as a targeted PDT agent.

Angiogenesis is an important process required for the development of new blood vessels, and is also crucial for tumorigenesis, tumor growth, survival, and metastasis. In the case of tumor-induced angiogenesis, transmembrane receptors such as integrins (*α*_v_*β*_3_ and *α*_v_*β*_5_) are highly expressed, which have a very high affinity for peptides containing RGD (Arg-Gly-Asp) and NGR (Asn-Gly-Arg) sequences. In this regard, complex **26** (Figure 10) with dual antiangiogenic and antitumor activity was a non-cytotoxic compound with IC_50_ >100 μM in different cancer cell lines (± *α*_v_*β*_3_ and *α*_v_*β*_5_ integrin receptors), while showing the antiangiogenic activity in HUVECs at sub-cytotoxic concentrations [89], which exemplified the design of angiogenesis inhibitors through conjugating a metallodrug with antiangiogenic activity to a cyclic RGD-containing peptide or a peptidomimetic analogue.

**Figure 11:**
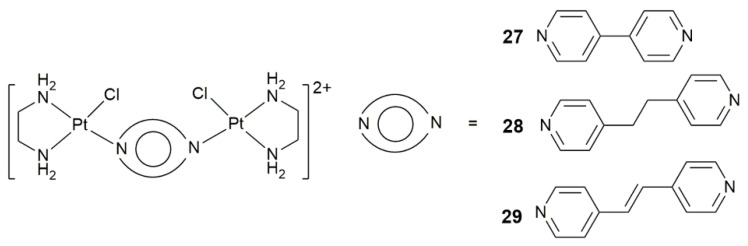
Chemical structures of complexes **27**–**29**.

Targeting angiogenesis provides an alternative direction for tumor-targeting therapy [90]. However, some complexes do not possess a specific targeting group, but still show antiangiogenic activity. For example, dinuclear complexes **27**–**29** (Figure 11) were found to interact with the phosphate backbone, forming Pt-DNA adducts with a minor groove covering [91]. These complexes, particularly complex **27**, are potential chemotherapeutics with anticancer and antiangiogenic activities, and no toxic effects at the desired concentration. They overcame cisplatin resistance in the zebrafish–mouse melanoma xenograft model and effectively blocked tumor neovascularization and melanoma cell metastasis. The activation of these complexes may result from their positive charge (+4) at the physiological conditions and affinity for DNA, heparan sulphate (HS), and enzyme heparanase (HPSE). It is worth noting that these complexes showed no sign of cardiovascular toxicity such as pericardial edema or disturbed heart beat rate, and liver toxicity such as liver necrosis, liver size change or reduced yolk absorption, which are the major obstacles limiting the long-term application of clinical anticancer drugs.

Organelle-targeting anticancer agents add a new dimension to the discovery and development of Pt drug candidates. Among different organelles, mitochondria have received much attention in recent years. The oxidative phosphorylation (OXPHOS) and glycolysis in mitochondria offer nutrients and energy to cancer cells for occurrence, growth, and transformation [92,93]. Nevertheless, unlike normal cells, most cancer cells preferentially use aerobic glycolysis as the metabolic pathway for glucose, which is accompanied by a high rate of glucose consumption and lactate production, even when oxygen is available for OXPHOS [94,95,96]. This abnormal energy metabolism process involves many proteins and enzymes, thus providing potential targets for the design of anticancer drugs and overcoming the drug resistance.

**Figure 12:**
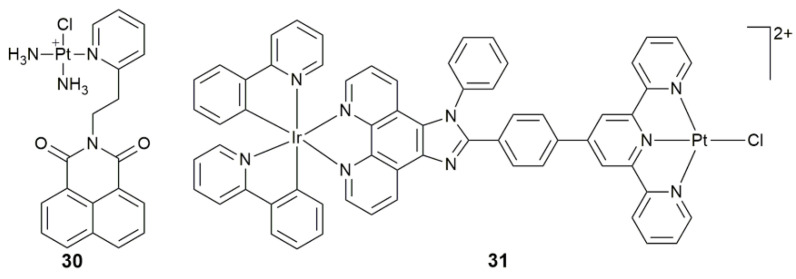
Chemical structures of complexes **30** and **31**.

It is generally believed that nDNA is the primary target of Pt-based anticancer drugs, and the resistance to Pt agents mainly result from the extensive repair of Pt-DNA adducts by the activation of DNA repair mechanisms in tumor cells [97,98,99]. Whereas, mitochondria contain their own cyclic mitochondrial DNA (mtDNA), which is more vulnerable to damage than nDNA due to the lack of histone protection and proximity to the ROS production site [100,101]. Therefore, mtDNA is a potential target for potentiating the activity of anticancer drugs. A cationic naphthalimide-modified complex **30** (Figure 12) not only caused severe nDNA damage but also induced the mtDNA lesion and regulated the downstream gene expression of mtDNA-encoded proteins [102]. Moreover, it disturbed the physiological process of mitochondria by reducing the mitochondrial membrane potential (MMP) and promoting the generation of ROS. Dinuclear complex **31** (Figure 12) was composed of Ir^III^ and Pt^II^ moieties. It accumulated in the mitochondria by a rate of up to 76% with an energy-independent uptake mechanism [103]. The complex exhibited strong antitumor activity towards A549R cisplatin-resistant cancer cells and damaged the mtDNA severely. Further, it disrupted the mitochondrial function, resulted in a loss of MMP, depleted ATP, and finally induced necrosis to cancer cells. All these findings suggest that mtDNA-targeted Pt complexes are potential antitumor agents against cisplatin-resistant cancer cells.

**Figure 13:**
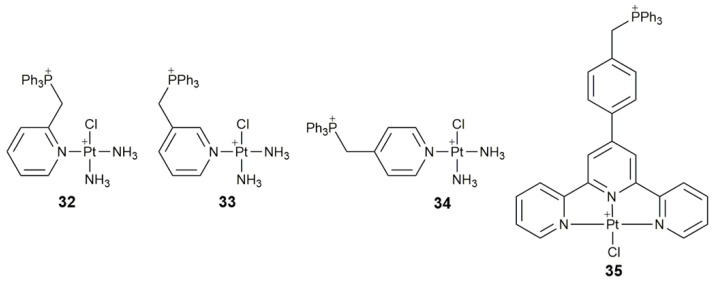
Chemical structures of complexes **32**–**35**.

Recently, we investigated the anticancer mechanism of three mitochondrion-targeted Pt^II^ complexes **32**−**34** (Figure 13) from the perspective of DNA damage, energy metabolism, and SAR [104]. Among them, complex **32** exhibited greater inhibitory activity on the A549 cells than cisplatin in vitro and vivo. Moreover, it not only combined with nDNA in a monodentate manner and damaged mtDNA, but also inhibited glycolysis of cancer cells, affected the structure and function of mitochondria, resulting in an abnormal process of mitochondrial OXPHOS and tricarboxylic acid cycle. In vivo studies showed that as the tumor shrank, the body weight of **32**-treated mice also reduced, which may be due to the mitochondrion-disrupting effect. Moreover, most of the Pt accumulated in the liver and kidneys, implying that the cellular uptake of **32** may be mediated by organic cation transporters, which are primarily expressed in these organs. This study provides new insights into the mechanism of action for Pt anticancer drugs.

Another mitochondrion-targeted complex **35** (Figure 13) that modified by triphenylphosphonium can modulate signaling pathways relevant to cancer bioenergetics [105]. It enhanced cytotoxicity against cisplatin-insensitive Caov-3 cells, exerted inhibition to mitochondrial thioredoxin reductase (TrxR), damaged mitochondrial morphology and function, destroyed both respiratory and glycolytic metabolisms, and induced cancer cells to enter into a hypometabolic state. The results highlight that targeting redox homeostasis and modulating metabolic pathways could effectively improve the anticancer effect.

**Figure 14:**
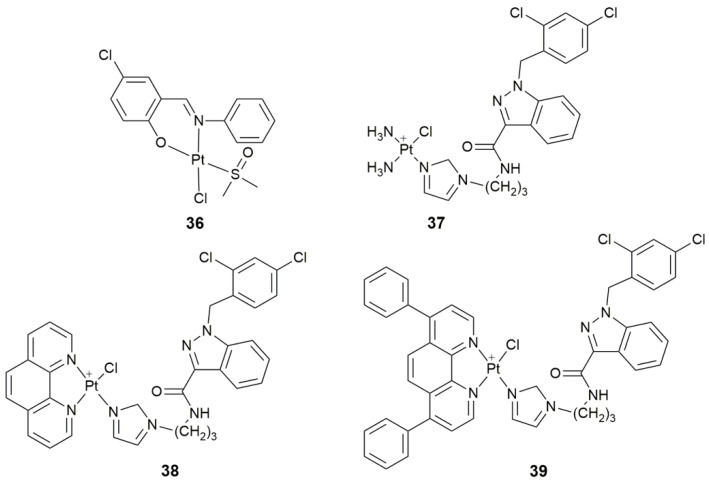
Chemical structures of complexes **36**–**39**.

In order to overcome the shortcomings of classic Pt drugs, extensive research has been initiated to search for new targets other than DNA. Enzymes play vital roles in almost all physiological and pathophysiological processes, and have long been considered as drug targets [106]. It is estimated that more than 47% of drugs target enzymes [107]. Therefore, enzyme inhibition could be a significant and alternative mechanism for Pt-based anticancer drugs. Protein tyrosine phosphatases (PTPs), a superfamily of enzymes, participate in the regulation of the intracellular signal transduction pathway by removing the phosphate groups from proteins [108]. Dysregulated activities of PTPs are related to the pathogenesis of many human diseases such as cancers, diabetes, and autoimmune diseases [108,109]. Complex **36** (Figure 14) displayed an antiproliferative activity against MCF-7 cells superior to cisplatin [110]. It selectively inhibited PTP1B, thus significantly influenced the cellular phosphorylation level and further the intracellular signal transduction pathway, which is distinctly different from the DNA-damaging mechanism for cisplatin, thereby providing a new clue for designing Pt-based anticancer drugs.

Hexokinase is the first rate-limiting enzyme in the glycolytic pathway, catalyzing the production of glucose-6-phosphate from glucose [111]. In normal cells, hexokinase isozymes have low transcriptional expression levels and each of them has tissue specificity, while as a key enzyme of glycolysis, hexokinase is widely and highly expressed in cancer cells, which not only promotes aerobic glycolysis, but also increases the resistance to cell death signals [112]. Complexes **37**−**39** (Figure 14), anchoring lonidamine (an inhibitor of hexokinase) to the Pt^II^ center, could selectively reduce the bioenergetics of cancer cells [113]. Particularly, **39** showed higher cytotoxicity than cisplatin against MDA-MB-231 cells (9.3 μM), caused significant damage to mtDNA, and disrupted mitochondrial bioenergetics. These complexes perturbed the signal pathways related to cell death, including DNA damage, the metabolic process, and transcription regulatory activity.

**Figure 15:**
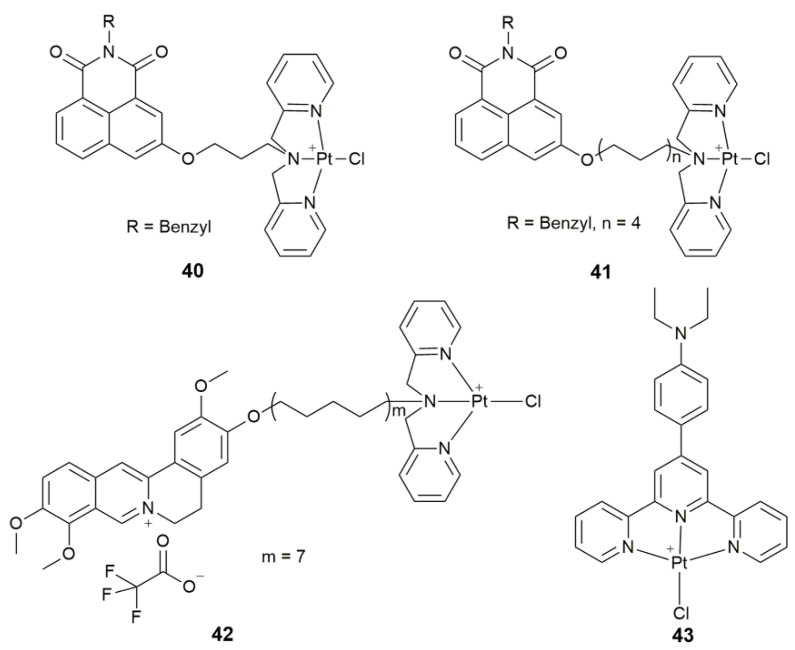
Chemical structures of complexes **40**–**43**.

Telomerase is present in the majority (85–90%) of cancer cells but is undetectable in normal cell lines, which is restricted by the level of hTERT and c-myc proteins [114,115]. Complexes **40**−**41** (Figure 15) induced apoptosis in the NCI-H460 cells via inhibiting the telomerase and disrupting the function of mitochondria at 0.89 and 0.10 μM, respectively [116,117]. In particular, **41** significantly inhibited the growth of tumor in NCI-H460 tumor-bearing mice with the tumor growth inhibition rate (TGI) of 40.7% and no obvious toxicity.

Complex **42** (Figure 15) containing a jatrorrhizine derivative also exhibited a remarkable antitumor activity and lower general toxicity in vitro and vivo compared to cisplatin [118]. It displayed high selectivity for HeLa cells (IC_50_ = 1.00 ± 0.17 nM) by targeting p53 and telomerase, and showed green luminescence. In addition, it caused mitochondrial and DNA damage, and induced a high rate of apoptosis even at a low dose of 1.00 nM. The HeLa tumor inhibition rate (TIR) of **42** (48.8%) was even higher than that of cisplatin (35.2%). The low systemic toxicity of **42** is quite impressive, in that the body weight of the treated mice (m_start_ = 18.6 ± 0.5 g, m_end_ = 20.1 ± 0.5 g) was hardly affected as compared with the control group (m_start_ = 18.7 ± 1.2 g, m_end_ = 20.7 ± 1.4 g). Similarly, complex **43** (Figure 15) exerted cytotoxicity mainly via inhibiting telomerase by interaction with the c-myc quadruplex and disruption of the mitochondrial function [119]. The complex exhibited selective cytotoxicity to T-24 cells.

## 5. Miscellaneous Monofunctinoal Pt^II^ Complexes

**Figure 16:**
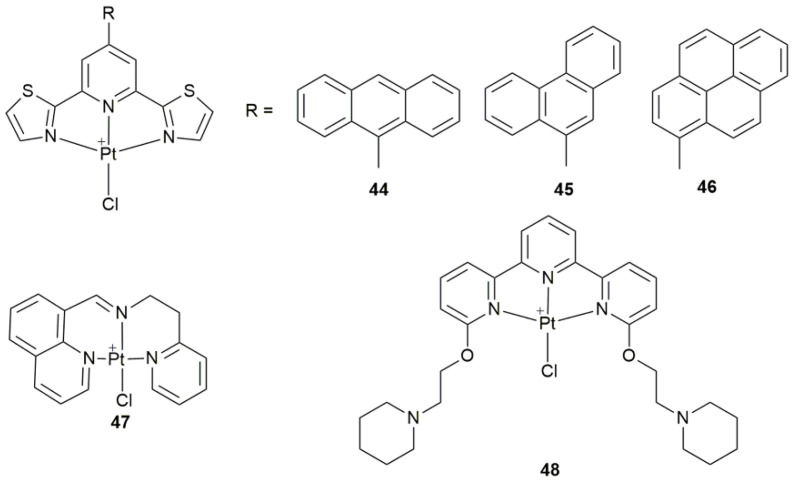
Chemical structures of complexes **44**–**48**.

Generally, Pt drugs induce cancer cell death by interfering with DNA synthesis or causing chemical damage to DNA, which is mainly manifested by apoptosis. However, accumulating evidences indicate that Pt drugs may have other molecular targets in addition to DNA, which can induce cell death through non-apoptotic pathways, such as autophagy, necrosis, and even immunogenicity [105,120,121,122,123]. Complexes **44**–**46** (Figure 16) showed a dose-dependent antiproliferative activity in the A2780 cells, with the cytotoxicity order of **44** < **45** < **46**, by a combinative apoptotic mechanism involving mitochondrial and autophagic pathways [124]. Complex **47** (Figure 16) not only initiated a series of events associated with mitochondrial dysfunction, but also induced an apparent ER stress through the ROS release and TrxR inhibition [125]. It simultaneously caused intrinsic pathway-dependent apoptosis and apoptosis-dependent pro-death autophagy in A549 cells. The interactions of **48** (Figure 16) with different topologies of DNA imply that it interacted with DNA non-covalently, but could degrade once reacted with proteins, forming adducts with different Pt/protein ratios [126]. This unusual mechanism of action may origin from the peculiar reactivity with biomacromolecules.

**Figure 17:**
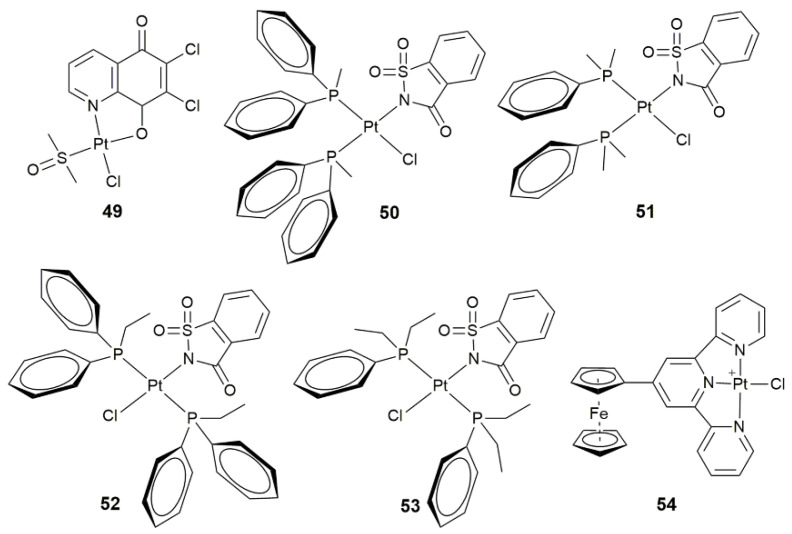
Chemical structures of complexes **49**–**54**.

Some monofunctional Pt^II^ complexes exhibit unique properties due to special structures. Complex **49** (Figure 17) could weaken the viability and invasibility of the human seminoma cells through the PI3K/Akt signaling and mitochondria-mediated apoptotic pathways [127]. It may serve as a potential drug in the treatment of testicular germ cell tumors. Complexes **50**−**53** (Figure 17) showed different biological activities owing to their different conformations, among which **50** and **51** with a cis configuration exhibited higher anticancer activity than **52** and **53** with a trans configuration towards cancer cells [128]. Concretely, complexes **50** and **51** showed a high affinity for the minor grooves of DNA, while **52** and **53** moderately bind to the major grooves of DNA. The enhanced anticancer activity of **50** and **51** may be attributed to their higher affinity for nDNA due to the formation of aqua species in the cell culture. All these complexes significantly increased the generation of ROS, which consequently depolarized the mitochondrial membrane and damaged the nDNA. Thus, the *cis*-complexes can be regarded as mitochondrial and DNA-targeting anticancer agents. In complex **54** (Figure 17), ferrocenyl terpyridine led to a dramatic decrease in the dark toxicity [129]. However, it showed a low lying broad absorption band at 600 nm and excellent ROS-mediated photocytotoxicity in visible light, with IC_50_ values of 9.5 and 12 μM in HaCaT and MCF-7 cell lines, respectively, which imply that **54** could act as an photoinitiator in visible light.

Considering the timeliness and novelty of the literature, the above mentioned compounds do not cover all the monofunctional Pt^II^ complexes and their research scope. If readers are interested in more details on the above complexes, please refer to the original papers and the following summary Table 1.

## 6. Conclusions

Platinum-based anticancer agents are the mainstay of chemotherapy regimens. Their drawbacks such as inherent or acquired drug resistance and systemic toxicity have stimulated the exploration of new possible drugs. Monofunctional Pt^II^ complexes are a potential new type of metallodrugs that break the traditional structure-activity relationships of platinum drugs and exhibit improved therapeutic efficacy. In this review, we introduced the basic conception of monofunctional Pt^II^ complexes and summed up some representative properties and potential applications. Fluorescent monofunctional complexes have the potentiality to monitor their distribution and travelling track in vitro and vivo with a high temporal and spatial resolution, which would help in understanding the therapeutic process of the complexes. Photoactive monofunctional complexes combine chemotherapy with photodynamic therapy, which provide a dual mechanism involving light-induced ROS and direct DNA damage to potentiate the action of PDT in hypoxic regions and overcome the drug resistance. Targeted monofunctional complexes could increase the amount of Pt content at the tumor site and avoid the side reactions with normal cells, thereby enhancing the efficacy and reducing the systemic toxicity of the complexes. All these characteristics have gone beyond the properties of existing platinum-based anticancer drugs. Simplicity in the synthesis in comparison with organic drugs is another advantage of monofunctional Pt^II^ complexes, or rather, all kinds of Pt complexes. Typically, there are three synthetic routes to these complexes. (1) For [Pt(NH_3_)_2_(Am)Cl]^+^ complexes, one chloride ion in cisplatin was first removed by silver nitrate or silver sulfate in dimethylformamide (DMF), and amine (Am) was then added to the solution. The goal product was obtained after the addition of diethyl ether or other organic solvents. (2) For [Pt(3Am)Cl]^+^ (Am = aromatic *N*-heterocyclic amine) complexes, a direct reaction between potassium tetrachloroplatinate(II) (K_2_PtCl_4_) and Am in dimethyl sulfoxide (DMSO) would give the target product. (3) Alternatively, *cis*-[Pt(DMSO)_2_(Cl)_2_] or bis(benzonitrile) dichloroplatinum(II) [Pt(PhCN)_2_Cl_2_] was mixed with Am in an organic solution (methanol or a mixture of methanol and acetonitrile/DMSO/acetone) to obtain the monofunctional Pt^II^ complex. If necessary, Am could be functionalized beforehand.

Last but not least, current researches on monofunctional Pt^II^ complexes are largely limited to the molecular and cellular levels, or at best to simple animal tests. Pharmacokinetic and clinical trial data are completely absent, which greatly hinder any objective assessment for the prospective development of these drug candidates. Recently, our studies indicate that some monofunctional Pt^II^ complexes do not react with DNA but still display anticancer activity [105,130]. The findings suggest that the known mechanism of action for these complexes is not impeccable, and many unknown facts need to be revealed in the future. In some less focused sides, the identification of molecular target and target-oriented molecular design, as well as the revealing of a new anticancer mechanism would be a meaningful aspect for the research of monofunctional Pt^II^ complexes.

## Figures and Tables

**Table 1:** Summary of monofunctional Pt^II^ complexes **1**−**54**.

Complex	Functional Group	Function	Tested Cells or Animals	Ref.
**Fluorescent Complexes**				
**1**	anthraquinone	monitor subcellular localization	U2-OS, U2-OS/Pt, A2780, A2780/DDP	[40]
**2, 3**	4-nitrobenzo-2-oxa-1,3-diazole	track cellular distribution	HeLa	[43]
**4**	4-amino-7-nitro-2,1,3- benzoxadiazole	in vitro and vivo fluorescence imaging	MCF-7, A549, 293T; zebrafish larva	[44]
**5**	ThT derivative	cellular imaging	HeLa	[45]
**Photoactive Complexes**				
**6, 7**	isomeric tetra-cationic(pyridyl)porphyrins	PDT on metastatic melanoma cells	WM1366	[56]
**8**	5,10,15,20-tetra-(4-pyridyl)-21*H*,23*H*-porphine	photocytotoxicity (50 W LED light, 6 J cm^−2^, 30 min)	colon26, sarcoma180; colon26 tumor-bearing mice	[57]
**9**	5,10,15,20-tetra(4-pyridyl)porphyrin	photocytotoxicity (6.95 J cm^−2^, 420 nm, 15 min), DNA photocleavage	MRC-5, HeLa, A2780, CP70	[51]
**10**	porphyrin containing Ga^III^ center	singlet oxygen generator, photocytotoxicity, DNA interaction	colon 26, sarcoma 180; colon26 tumor-bearing mice	[59]
**11**	Si^IV^ phthalocyanine	specific cellular uptake, mitochondrial accumulation, photocytotoxicity (45 min, 660–680 nm, 5.5 ± 2.5 mW cm^−2^)	MDA-MB-231, HEK293T	[62]
**12, 13**	Si^IV^ phthalocyanine	photocytotoxicity, DNA-targeting PDT agents	HeLa	[63]
**14, 15**	4,4-difluoro-4-bora-3a,4a-diaza-*s*-indacene (BODIPY) and its diiodo derivative	photocytotoxicity (400−700 nm, 10 J cm^−2^), cellular imaging	HaCaT, MCF-7	[64]
**16**	*α*-(4-amino)styryl-4,4-difluoro-4-bora-3a,4a-diaza-*s*-indacene	photocytotoxicity (532 nm, 3.5 mW cm^−2^, 5 min)	MCF-7, SGC-7901, A549, HeLa	[66]
**17**	*α*-(4-amino)styryl-4,4-difluoro-4-bora-3a,4a-diaza-*s*-indacene	photocytotoxicity, lysosomal escape, increase accessibility to nDNA, decrease intracellular GSH	MCF-7, SGC-7901, A549, HeLa	[70]
**18–21**	triphenylamine core	PDT activity	HeLa, HepG2, A549, A549cisR, LO2; HeLa xenograft-bearing nude mice	[76]
**Targeted Complexes**				
**22–23**	bisphosphonate	bone targeting	U2-OS, MG-63; male ICR mice	[84]
**24**	bisphosphonate	bone targeting, decrease systemic toxicity	U2-OS, MG-63, LO2; male ICR mice	[85]
**25**	c(RGDyK)	tumor targeting, integrin-targeted PDT	SKOV-3, PC-3, A549, MCF-7, MDA-MB-231, U87M	[88]
**26**	cyclic peptide containing RGD sequence (-Arg-Gly-Asp-)	target angiogenesis, antiangiogenic and antitumor activity	SK-MEL-28, MDA-MB-231, CAPAN-1, HUVEC	[89]
**27–29**	4,4’-bipyridine, 1,2-di(pyridin-4-yl)ethane, or 1,2-di(pyridin-4-yl)ethene	target angiogenesis, overcome cisplatin resistance, block tumor neovascularization and metastasis	MRC-5, A549, A375; B16-F10 melanoma-zebrafish, HCT-116-zebrafish	[91]
**30**	naphthalimide	target mtDNA, damage mtDNA, regulate mtDNA-encoded protein, disturb mitochondrial physiological process	MCF-7, A549, Caov-3, HK-2; MCF-7 tumor-bearing mice	[102]
**31**	Ir^III^ moiety plus imidazo[4,5-f][1,10]phenanthroline derivative	target mtDNA, accumulate in mitochondria, induce mitochondrial dysfunction via mtDNA damage	HepG2, HeLa, A549, A549R	[103]
**32–34**	triphenylphosphonium	target mtDNA, inhibit glycolysis, affect mitochondrial structure and function, damage mtDNA	A549, HeLa, SMMC, HL-7720; A549 tumor-bearing mice	[104]
**35**	triphenylphosphonium	target mitochondrion, inhibit mitochondrial TrxR, destroy respiratory and glycolytic metabolisms	Caov-3, A549, A549R, HK-2	[105]
**36**	5-chlorosalicylideneaniline	target tyrosine phosphatases, selectively inhibit PTP1B, antiproliferative activity	MCF-7, HepG2, A549	[110]
**37–39**	lonidamine	target hexokinase, disrupt mitochondrial bioenergetics, damage mtDNA	A549, PC3, Caov-3, MCF-7, MDA-MB-231, MCF-10A	[113]
**40, 41**	naphthalene imide derivatives	target telomerase, inhibit telomerase, disrupt mitochondrial function	SKOV-3, NCI-H460, HeLa, HL-7702, BEL-7402; NCI-H460 tumor-bearing mice	[116,117]
**42**	jatrorrhizine derivative	target telomerase and p53, cause mitochondrial and DNA damage, display antitumor activity and green luminescence	SKOV-3/DDP, T-24, HeLa, HL-7702, A549; HeLa tumor-bearing mice	[118]
**43**	4-([2,2′:6′,2′′-terpyridin]-4′-yl)-N,N-diethylaniline	target telomerase, inhibit telomerase by interacting with c-myc quadruplex, disrupt mitochondrial function	BEL-7404, A549, MGC80-3, T-24, HL-7702	[119]
**Miscellaneous Complexes**				
**44–46**	9-anthryl, 9-phenantryl, and 1-pyrenyl 2,6-bis(thiazol-2-yl)pyridines	participate in apoptotic mechanism involving mitochondrial and autophagic pathways	MCF-7, PC3, HCT-116, A2780, Fibroblasts	[124]
**47**	8-substituted quinoline derivatives	induce ER stress, cause apoptosis and pro-death autophagy	BEL-7404, SKOV-3, HepG2, HCT-116, HL-7702; A549 tumor-bearing mice	[125]
**48**	terpyridine with two piperidine substituents	peculiar reactivity with biological macromolecules (proteins)	hen egg white lysozyme (HEWL, protein)	[126]
**49**	6,7-dichloro-5,8-quinolinedione	induce apoptosis via PI3K/Akt signaling and mitochondria-mediated apoptotic pathways	TCam-2, SEM-1	[127]
**50–53**	mono- and dialkylphenylphosphines	conformation-dependent biological activity	MCF-7, A549, BEAS-2B, HCT-116	[128]
**54**	ferrocenyl-terpyridine	photocytotoxicity in visible light (400–700 nm)	HaCaT	[129]

## Appendix A

**Table A1:** Designations of cell lines.

Codes	Designated Cell Lines
A2780; CP70	human ovarian cancer cell
A2780/DDP	cisplatin-resistant human ovarian cancer cell
A375	human melanoma cell
A549	human lung carcinoma cell
A549cisR; A549R	cisplatin-resistant human lung carcinoma cell
B16-F10	mouse melanoma cell
BEAS-2B	human normal bronchial epithelial cell
BEL-7402	human hepatoma cell
Caov-3	human ovarian cancer cell
CAPAN-1	human pancreatic adenocarcinoma cell
colon26	murine colon carcinoma cell
HaCaT	human keratinocyte
HCT-116	human colon carcinoma cell
HEK293T; 293T	human embryonic kidney cell
HeLa	human cervical carcinoma cell
HepG2	human hepatocarcinoma cell
HK-2	human renal tubular epithelial cell
HL-7702; LO2	human normal liver cell
HUVEC	human umbilical vein endothelial cell
MCF-10A	human normal breast epithelial cell
MCF-7	human breast adenocarcinoma cell
MDA-MB-231	human breast cancer cell
MG-63	human osteosarcoma cell
MGC80-3	human gastric adenocarcinoma cell
MRC-5	human normal lung fibroblast cell
NCI-H460	human lung carcinoma cell
PC-3; PC3	human prostate cancer cell
sarcoma180	mouse sarcoma cell
SEM-1	human testicular seminoma cell
SGC-7901	human gastric adenocarcinoma cell
SK-MEL-28	human melanoma cell
SKOV-3	human ovarian cancer cell
SKOV-3/DDP	cisplatin-resistant human ovarian cancer cell
SMMC	human hepatocellular carcinoma cell
T-24	human bladder cancer cell
TCam-2	human testicular seminoma cell
U2-OS	human osteosarcoma cell
U2-OS/Pt	cisplatin-resistant human osteosarcoma cell
U87M	human glioblastoma cell
WM1366	human melanoma cell
